# A comparison of machine learning algorithms for the surveillance of autism spectrum disorder

**DOI:** 10.1371/journal.pone.0222907

**Published:** 2019-09-25

**Authors:** Scott H. Lee, Matthew J. Maenner, Charles M. Heilig

**Affiliations:** Centers for Disease Control and Prevention, Atlanta, GA, United States of America; Indiana University Purdue University at Indianapolis, UNITED STATES

## Abstract

**Objective:**

The Centers for Disease Control and Prevention (CDC) coordinates a labor-intensive process to measure the prevalence of autism spectrum disorder (ASD) among children in the United States. Random forests methods have shown promise in speeding up this process, but they lag behind human classification accuracy by about 5%. We explore whether more recently available document classification algorithms can close this gap.

**Materials and methods:**

Using data gathered from a single surveillance site, we applied 8 supervised learning algorithms to predict whether children meet the case definition for ASD based solely on the words in their evaluations. We compared the algorithms’ performance across 10 random train-test splits of the data, using classification accuracy, F_1_ score, and number of positive calls to evaluate their potential use for surveillance.

**Results:**

Across the 10 train-test cycles, the random forest and support vector machine with Naive Bayes features (NB-SVM) each achieved slightly more than 87% mean accuracy. The NB-SVM produced significantly more false negatives than false positives (*P* = 0.027), but the random forest did not, making its prevalence estimates very close to the true prevalence in the data. The best-performing neural network performed similarly to the random forest on both measures.

**Discussion:**

The random forest performed as well as more recently available models like the NB-SVM and the neural network, and it also produced good prevalence estimates. NB-SVM may not be a good candidate for use in a fully-automated surveillance workflow due to increased false negatives. More sophisticated algorithms, like hierarchical convolutional neural networks, may not be feasible to train due to characteristics of the data. Current algorithms might perform better if the data are abstracted and processed differently and if they take into account information about the children in addition to their evaluations.

**Conclusion:**

Deep learning models performed similarly to traditional machine learning methods at predicting the clinician-assigned case status for CDC’s autism surveillance system. While deep learning methods had limited benefit in this task, they may have applications in other surveillance systems.

## Introduction

The Centers for Disease Control and Prevention (CDC) coordinates a labor-intensive process to measure the prevalence of autism spectrum disorder (ASD) among children in the United States. Maenner et al.[1] developed a promising machine learning approach that could assist with portions of this process. In this paper, we expand on this initial work by evaluating a wider variety of machine learning models.

ASD (here used interchangeably with “autism”) refers to a group of heterogeneous neurodevelopmental conditions characterized by impairments in social interaction and the presence of repetitive behaviors or restricted interests. ASD is diagnosed through the observation of behavior consistent with the criteria described in the *Diagnostic and Statistical Manual of Mental Disorders*.[2] Since 2000, CDC has monitored the prevalence of ASD among US children in selected communities through the Autism and Developmental Disabilities Monitoring (ADDM) Network using a process by which trained clinicians review children’s medical and educational evaluations to identify behaviors consistent with the DSM criteria for ASD. The surveillance case definition, which serves a different purpose than a medical diagnosis, allows the ADDM Network to identify children who have descriptions of the requisite behavioral features documented in their records, but do not necessarily have an ASD diagnosis. Every two years, the ADDM Network has used this method to estimate the prevalence of ASD in 8-year-old children, ranging from 1 in 150 children in 2000 to 1 in 68 children in 2012. The ADDM network has yielded crucial insights into the epidemiology of ASD in terms of understanding prevalence, disparities in diagnosis, and the contribution of risk factors to the changes in prevalence over time.

Because expert clinicians must manually review each child’s evaluations to determine whether they meet the surveillance case definition, the system is both labor-intensive and time-consuming. To explore ways of making the review process more efficient, Maenner et al.[1] developed a machine learning algorithm for automatically determining whether children meet the ADDM surveillance case definition for ASD based solely on the text contained in their written evaluations. By training a random forest[3] on written evaluations collected in 2008, they were able to predict classifications for evaluations collected in 2010 with good diagnostic accuracy, achieving an F_1_ score of 86.6% and an accuracy score of 86.5%, compared to interrater agreement among the expert reviewers of 90.2%[4]; accuracy and F_1_ are further explained in the methods section. The algorithm could also be used by ADDM clinicians to screen children during the manual review process and to focus their efforts on cases that are harder to classify, where good judgment and clinical experience are critical for classification.

We conceived our study to expand upon Maenner et al.’s random forest analysis in order to achieve three primary goals. First, we sought to determine whether we can achieve higher accuracy on the case classification task using more recently available analytical methods, including those falling under the broad umbrella of deep learning. Second, we wanted to assess the variability in performance of these methods, as Maenner et al. considered only 1 train-test split. Finally, we aimed to compare differences in the prevalence estimates produced by these methods, which has direct bearing on their suitability for surveillance. We discuss ways in which these models may be used effectively to enhance autism surveillance.

## Methods

### ADDM overview

Maenner et al.[1] provide a detailed overview of the ADDM Network, including the labor-intensive review process used to determine whether children meet the surveillance case definition for ASD, and an explanation of how machine learning algorithms may be used to assist clinicians in conducting the manual reviews. In brief, each site in the network requests to review medical records for children evaluated for having developmental disabilities and educational records for children served in a special education program. These records are screened by ADDM Network staff. If a record contains a possible indication of autism (including a diagnosis, specific behaviors, and if a comprehensive autism evaluation was performed), the text from the child’s developmental evaluations are extracted into the surveillance database. Evaluations from multiple sources are combined into a single, de-identified record and reviewed by trained clinicians to determine if they meet the ADDM Network ASD case definition. Because the focus of this study is on the comparison of methods for document classification rather than their implementation in the ADDM surveillance workflow, we refer readers to other sources[5,6] for more information on the structure and goals of the network.

### Corpus and data structure

Our dataset consists of the abstracted evaluations and corresponding surveillance case classifications for all children evaluated in years 2006[7], 2008[4], and 2010[8] at the Georgia ADDM site. During these 3 waves, 3,379 children were reviewed at the site, among which 1,829 (48.9%) met the ADDM surveillance case definition for ASD. Our analytic dataset is a corpus *D* of 3,739 documents, with a vocabulary size *V* of 59,660 and a total word count *W* of 7,845,838. The documents range in length from only a couple of words to the tens of thousands (Table 1). We briefly discuss the effect this variability may have on classification accuracy in the supplement.

**Table 1:** Minimum, first quartile, median, third quartile, and maximum word counts per child. The first row shows statistics for total (i.e., non-unique) words, while the second shows those for unique words. We represented each child’s record as the unordered collection of his or her abstracted evaluations, which we treated as a single block of text (i.e., a document). We preprocessed the text by lowercasing all strings, removing stop words and special characters, and converting all words to their dictionary forms, or lemmas.

	Min	1Q	Med	3Q	Max
*Total*	2	813	1,528	2,737	20,801
*Unique*	2	344	527	773	2407

For our baseline classifiers, we represented each child’s collection of abstracted evaluations as a single document in a bag-of-words (BoW) model. Under this model, each document *d* is represented as a row vector of word counts, where each entry in the row corresponds to the number of times a particular word *w* appears in the document. The entire corpus is represented as a *d* x *V* document-term matrix, where each row is the BoW vector for a particular child's combined abstracted evaluations. To make our classifiers more effective, we counted both single words, or unigrams (n = 59,660), and pairs of adjacent words, or bigrams (n = 830,803); this yielded a total of 890,463 features in our data representation. BoW classification models are computationally efficient and readily applied using widely available, open-source software. In addition, some classifiers applied to BoW data can yield metrics interpretable as feature importances, which can give investigators useful clues as to how the model learns to discriminate cases from non-cases.

### Description of classifiers

We compared several baseline classifiers to the random forest algorithm published by Maenner et al.[1]: latent Dirichlet allocation (LDA)[9,10]; latent semantic analysis (LSA)[11]; multinomial naive Bayes (MNB)[12]; support vector machine (SVM) with a linear kernel[13]; interpolated naive Bayes-SVM (NB-SVM)[14,15]; and two neural networks adapted from the fastText architecture[16].

Latent Dirichlet allocation (LDA)[9] is an unsupervised algorithm typically used for topic modeling rather than document classification. LDA models documents as mixtures of topics, which themselves are modeled as mixtures of words, allowing it to model complex and often subtle information in large collections of text. LDA has been adapted with some success for supervised learning problems.[10] We consider LDA as a way to generate dense vector representations of the evaluations, which can then be used as input for training a supervised algorithm. Latent semantic analysis[11] is a dimensionality reduction technique that generates dense representations of the evaluations by applying a singular value decomposition to the document-term matrix. For both LDA and LSA, we used a linear SVM [13] to perform the case classification task.

Multinomial Naive Bayes (NB)[12] is a supervised learning algorithm that uses Bayes’ rule to calculate the probability that a document belongs to a certain class based on the words (also known as features) that it contains, under the assumption that the features are statistically independent conditional on class membership. It is often used as a baseline model for text classification. Multinomial NB produces the most likely features for each class of documents, which can yield keywords associated with evaluations meeting the surveillance case definition for ASD. The model can also generate predicted class probabilities to use for classification.

While multinomial NB models are interpretable and quick to train, they have some formal shortcomings,[12] like the conditional independence assumption mentioned above, and they are often outperformed by discriminative models like the support vector machine (SVM). For this reason, we also included 2 versions of the SVM using the document-term matrix as input: a simple linear-kernel SVM, and an interpolated Naive Bayes-SVM (NB-SVM).[14] The SVM constructs a maximum-margin decision boundary between document classes based on the original document-term matrix. The NB-SVM constructs a decision boundary using NB features, which makes it competitive with state-of-the-art models for sentiment analysis.[14] The model tends to work best when nonzero word counts are converted to 1, or binarized. This change makes the weights in the trained model heuristically (but not strictly) interpretable as a kind of feature importance.

Our final models are both neural networks and are simplified versions of the fastText architecture.[16] Like the NB-SVM, they take a binarized document-term matrix as their input, and like a traditional logistic regression model, they output class probabilities via the softmax function that can be used for document classification. Unlike the other models in our experiments, the networks feature an embedding layer between the input and softmax layers, allowing them to learn dense vector representations of words rather than documents. In the original fastText architecture, these vectors are averaged to generate document representations; we reuse this method for our first model (NN_avg_), and we replace the averaging layer with a summation layer for our second (NN_sum_).

A supplement provides additional technical details about the model architectures, hyperparameters, and implementation.

### Hyperparameter optimization

Before performing our experiment, we randomly split the full dataset into a training set and a validation set, which we then used to select hyperparameter values for each model. We used a variety of methods for tuning, including grid search (LSA and LDA), a combination of non-recursive and recursive feature elimination (the random forest), and a Bayesian optimization procedure based on Gaussian processes (all other models). We provide detailed descriptions of the optimization procedure for each model in the Supplement.

### Experimental setup

Maenner et al.[1] mimicked real-world conditions by training their model on data gathered from the Georgia ADDM site in 2008 and then testing it on data collected in 2010. Because we were interested in assessing both the performance and the variability in performance of our models, we formulated our experiment as a series of 10 train-test cycles, where the training data are selected randomly from the entire dataset rather than by year. For each of these cycles, we randomly split the entire dataset into 57% training, 13% validation, and 30% test sets. We then fit each model to the training data, and measured its performance on the test data using common measures of binary classification accuracy, including raw accuracy (the proportion correctly classified) and F_1_ score (the harmonic mean of sensitivity and positive predictive value). Because public health surveillance relies on accurate prevalence estimates, we also measure the difference between each model’s number of positive calls and the true number of cases in the test set. We used a fixed list of 10 seeds for the random number generator to ensure that the models were fit and tested on exactly the same data splits.

To assess the performance of the models across the 10 train-test splits, we focused on two metrics: mean classification accuracy (individual-level prediction), and mean difference in prevalence from the true prevalence in the test data (population-level prediction). For each metric, we selected the model with the highest accuracy or discordance closest to 0 as the referent. We constructed simultaneous 95% confidence intervals by applying Dunnett’s procedure for multivariate normal distributions to control the family-wise error rate (FWER) for multiple comparisons.

### Technical notes

The LDA, LSA, multinomial NB, SVM, and random forest models were implemented in Python using scikit-learn v0.19,[17] which was also used to preprocess the text and generate the document-term matrices. The NB-SVM was implemented in NumPy,[18] with the SVM component imported from scikit-learn, and the neural networks were implemented in Keras with the TensorFlow backend.[19] Bayesian hyperparameter optimization was implemented using GPyOpt. Finally, statistical analysis was conducted in R 3.5.1 [20], and the simultaneous confidence intervals being constructed using the multcomp package [21].

This analysis was submitted for human subjects review and deemed to be non-research (public health surveillance) according to CDC policy.

### Data availability

The primary data in this analysis are medical and educational evaluations collected for public health surveillance under an assurance of confidentiality pursuant to the Public Health Service Act, §308(d). Due to the sensitive nature of these documents, we will make these data available (upon request) in the form of the final term-document matrices used to train and test the models’ performance rather than the raw text of the evaluations; these matrices will not include an enumeration of the *n*-grams associated with the features, and so they will be purely numeric. CDC’s National Center on Birth Defects and Developmental Disabilities requires a signed data use agreement by anyone requesting data from the Metropolitan Atlanta Developmental Disabilities Surveillance Program (MADDSP) to ensure that: 1) the data are analyzed for the specific purpose of the proposal submitted, and 2) the investigator will not try to identify any child or present stratified analyses leading to a sample <5 children. These two points are what result in the dataset being considered a restricted public use dataset. All requests for MADDSP public use datasets should be submitted to ncbddddata@cdc.gov.

### Code availability

The code for our models, optimization procedures, and experiments is available on GitHub at https://github.com/scotthlee/autism_classification/.

## Results

We present the mean binary classification metrics for each of our models across the 10 train-test splits in Table 2.

**Table 2:** Mean performance for our 8 models across the 10 train-test splits. Metrics include sensitivity (Sens), specificity (Spec), positive predictive value (PPV), negative predictive value (NPV), F_1_, and accuracy (Acc), all shown as percentages. The best scores for each metric are shown in bold, and the final column presents differences in accuracy between each of the models and the most accurate model, the NB-SVM. Simultaneous confidence intervals are multiplicity-adjusted to control FWER.

Model	Sens	Spec	PPV	NPV	F_1_	Acc (95% CI)	Diff acc (95% CI)
LDA	44.2	72.4	60.6	57.5	51.1	58.6 (55.0, 62.2)	-29.0 (-32.4, -25.6)
MNB	82.3	72.6	74.2	81.0	78.0	77.3 (73.9, 80.7)	-10.3 (-12.5, -8.1)
SVM	83.5	84.5	83.8	84.2	83.6	84.0 (80.8, 87.2)	-3.7 (-6.6, -0.7)
LSA	81.5	88.5	87.2	83.3	84.2	85.1 (83.1, 87.0)	-2.6 (-4.2, -0.9)
NN_sum_	85.5	84.7	84.4	86.0	84.9	85.1 (81.9, 88.3)	-2.6 (-5.2, 0.1)
NN_avg_	86.3	86.4	85.9	86.9	86.0	86.3 (84.4, 88.2)	-1.3 (-3.3, 0.6)
RF	**87.0**	87.1	86.6	**87.5**	86.8	87.1 (83.8, 90.4)	-0.5 (-2.2, 1.1)
NB-SVM	85.2	**89.9**	**89.0**	86.4	**87.1**	**87.6** (85.2, 90.1)	*

The NB-SVM achieved the highest mean accuracy (87.6%) across the 10 train-test cycles, followed by the random forest (87.07%), the averaging neural network (86.3%), and the summing neural network (85.08%). The mean F_1_ scores were also very close, with the top 2 models, the NB-SVM (87.1%) and the random forest (86.8%), being separated by only a quarter of a percentage point; these 2 models also achieved the highest scores for sensitivity, specificity, PPV, and NPV. Although five models achieved accuracy of over 85%, the random forest and the two neural networks were the only models whose accuracy did not significantly differ from the NB-SVM.

Although our classifiers yielded similar accuracy, they differed in their proportions of positive calls, as well as in the distribution of their incorrect predictions between positive and negative calls. The random forest and the two neural networks produced about as many false positives (FPs) as false negatives (FNs), with mean proportions positive of 48.4% and 48.7% respectively (Table 3).

**Table 3:** Mean prevalence-related metrics for our 8 models across the 10 train-test splits. Metrics included are false positives (FP); false negatives (FN); number of positive calls (Pos calls); number of true positives in the test set; discordance; and the difference in discordance from the least discordant model, the SVM. Here, discordance is the difference between the predicted percentage positive and the true percentage positive. Simultaneous confidence intervals are multiplicity-adjusted to control FWER.

Model	FP	FN	Pos calls	True pos	Disc % (95% CI)	Diff disc % (95% CI)
LDA	158	306	401	549	-13.2 (-15.1, -11.3)	-13.0 (-15.5, -10.5)
MNB	157	97	609	549	-3.2 (-5.1, -1.3)	5.5 (1.3, 9.8)
LSA	66	102	513	549	-2.1 (-5.5, 1.4)	-3.0 (-5.2, -0.9)
NB-SVM	58	81	526	549	-2.0 (-5.4, 1.3)	-1.9 (-5.5, 1.7)
RF	74	71	552	549	-1.1 (-3.5, 1.4)	-0.9 (-3.7, 1.9)
NN_sum_	88	80	557	549	0.7 (-2.6, 4.0)	0.9 (-2.3, 4.1)
NN_avg_	78	76	552	549	0.3 (-2.9, 3.5)	0.4 (-3.0, 3.9)
SVM	89	91	547	549	**-0.2 (-3.4, 3.0)**	*

The NB-SVM and LSA models, however, leaned more heavily on FNs than FPs, with mean differences of -23 and -36 respectively in the number of positives from the test set. On the other hand, MNB produced many more FPs than FNs, resulting in a higher mean prevalence estimate than the true percentage positive. Overall, the only models to produce estimates that were significantly different from the true percentage positive were the LDA and MNB models, which under- and over-estimated the percentage, respectively.

## Discussion

Our baseline models are strong to enough enhance the current surveillance workflow: Their accuracy is within 5% of human levels on the same task, they are computationally feasible, and they are heuristically interpretable. As we discuss here, more sophisticated models alone cannot be expected to improve performance without enriching the representation of the data, e.g., by way of feature engineering, richer representation of text than unigram and bigram bag-of-words, or including other information from the children’s records in the model.

Perhaps our most important result is that the random forest was among the models that were statistically indistinguishable from the NB-SVM in its individual-level performance (i.e. its classification accuracy) and from the SVM in its population-level performance (i.e. its prevalence estimate). Given the interpretability of its feature importances, these two results suggest that the random forest stands out as a good candidate for surveillance applications among the models that we evaluated.

For surveillance purposes, accuracy or F_1_ scores may have less practical importance than the number of positive calls, which public health practitioners often use to generate model-based prevalence estimates. In a fully-automated workflow, then, the random forest or neural network may be more appropriate for conducting surveillance, since they produce more accurate prevalence estimates without sacrificing much in the way of individual-level predictive quality. As a bonus, these two methods also naturally produce predicted class probabilities, which could be used to support the current surveillance workflow by helping clinicians focus on evaluations that may be particularly hard to classify. In a partially-automated setting, however, the NB-SVM may still be useful as a support tool for clinicians conducting a manual review of the written evaluations, especially if cross-validation (e.g., by way of Platt scaling[22]) is used to obtain non-thresholded probability estimates that are well-calibrated to the true distribution of class labels.

Another important result is that none of the models was able to match the levels of interrater agreement seen in the ADDM network’s ongoing quality reviews[4], although both the random forest and the NB-SVM achieved over 89% on several train-test cycles. In broad terms, this result suggests that the clinicians reviewing the evaluations rely on more than just the text they contain to determine whether a child meets the surveillance case definition for ASD. In practice, the ADDM clinicians have access to more than just the written evaluations when making their case classifications. Because interrater agreement among the clinicians hovers around 91%, we would likely need to add extra features to the analytic data to lower the error rate, regardless of which document-level classifier is used. Maenner et al.[1] made note of this in their original analysis, noting three possible refinements beyond document-level models to improve classification: (1) accounting for characteristics of each child’s set of evaluations (such as total number and mix of school or health sources), (2) adding phrase-level information to the document-level classifiers to approximate the symptom-based scoring rubric used by the clinicians, or (3) using additional characteristics of the children themselves, such as sex or IQ. Since our purpose in this analysis was to compare alternative document-level classifiers, we did not assess the potential incremental improvements from using other features. Based on our results, we conclude that using additional features would be the logical way to further reduce the gap between a machine algorithm and clinical interrater agreement.

To address the question of whether more sophisticated text-classification models could achieve higher levels of accuracy on this particular task, we refer back to the child-level descriptive statistics for the corpus (Table 1), which demonstrate two important characteristics of the ADDM dataset: variability in length of the abstracted evaluations, and variability of their vocabularies. The BoW model is able to accommodate this variability in a straightforward way, through the construction of the document-term matrix and its variants, but it may pose a challenge for other classification models. Recurrent neural networks (RNNs) can have a hard time learning long sequences due to the vanishing/exploding gradient problem.[23] Long short-term memory networks[24] and gated recurrent units[25] generally solve this problem by altering the standard RNN cell so that it forgets information that is unimportant for prediction, would be unlikely to classify the longest documents in our dataset without substantial modification. Convolutional neural networks (CNNs) have also been used for text classification,[26,27] but they do not appear to work well for longer chunks of text without substantial modification. Denil et al.[28] used a hierarchical CNN to generate document representations from lower-level information in the text, like words and sentences. These and other sophisticated models, like a recurrent CNN,[29] a gated recurrent network,[30] and paragraph vectors[31] may achieve higher levels of classification accuracy on this particular task. They may not be worth the effort to implement, however, given our results. Our baseline classifiers have simpler architectures, are far less computationally intensive, and produce relatively unbiased prevalence estimates, all without sacrificing much in the way of individual-level prediction.

On a practical note, public health practitioners should carefully consider what they hope to achieve by applying machine learning to surveillance, and they should choose models that will help them achieve these specific goals. In low-resource settings where continuing expert review is infeasible and the model alone will be used to generate prevalence estimates, diagnostic accuracy may be less important than similarity between the proportion of positive calls produced by the model and the class labels in the actual data. Statistical methods for paired proportions, like McNemar’s test or Newcombe’s[32] method for estimating the corresponding confidence intervals, can be used in these contexts to judge the quality of predictions.[33,34] In higher-resource settings where expert review is a component of the surveillance system, as in the ADDM network, probabilistic calibration through measures like the Brier score or cross-entropy loss becomes more important, since reviewers can use the model-based probability estimates to focus their efforts on cases that are hard to classify. Sensitivity, specificity, and other measures of binary diagnostic accuracy are still useful, especially when models are used for patient-level screening or diagnosis, but when models are used for population-level surveillance, the other measures bear careful consideration.

## Conclusion

Although more sophisticated models do not appear to be necessary for improving the autism surveillance workflow, these and other deep models could be useful in the general sense for other public health applications; CDC, for example, maintains several large databases containing unstructured text for which these methods might improve the efficiency of surveillance systems.

## Supporting information

S1 File Supplemental methods. (DOCX)
