# Supplementary material for: A comparison of machine learning algorithms for the surveillance of autism spectrum disorder
Source: PLoS One. 2019 Sep 25;14(9):e0222907. doi: 10.1371/journal.pone.0222907 (PMC6760799; doi:10.1371/journal.pone.0222907)
Supplement: S1 File — (DOCX) [file pone.0222907.s001.docx]

**S1 File. Supplemental methods**

**A. Effect of document length on classification accuracy**

Here we explore the relationships between document length, measured as the total number of words in a child’s collected evaluations, and classification accuracy. The plot below shows locally-estimated scatterplot smoothing (LOESS) curves for each of our models across the 10 train-test splits, with the vertical bars at the top and the bottom of the plot indicating children who did and did not meet the surveillance case definition for ASD, respectively.

We note that the probability of meeting the case definition increases with the number of words in the evaluations, perhaps because children with autism are more likely to undergo extensive evaluation and care than those without, or perhaps because the ADDM network clinicians are less likely to make positive calls with less information at hand than with more. In terms of model performance, we see that for short documents, classification accuracy is highly variable—some models, like the averaging neural network and the random forest, do especially well, while others, like the SVM and the MNB classifier, do poorly. This gap narrows as document length increases, however, and once the median document length is reached (median=1,528 words), accuracy improves with length for all models, showing that they perform reasonably well even at the extreme end of the range, e.g., for documents with more than 10,000 words.

**B. Model architectures and hyperparameter optimization procedures**

*B0. Overview of Hyperparameter Optimization Procedures*

After splitting the data randomly into a training set and a validation set (note: the seed for this split was not used as a seed for any of the 10 train-test splits reported in the primary experiment), we use 3 kinds of optimization procedures to tune the hyperparameters for our models: grid search (LSA and LDA); recursive feature elimination (RFE; random forest); and a Bayesian method for Gaussian process optimization (GPO; all other models). We implement grid search using NumPy; RFE using a combination of NumPy and scikit-learn; and GPO using the GPyOpt library for Python. All GPO used the default parameters for the methods.bayesian_optimization module in GPyOpt, but we changed the number of iterations for which the process was allowed to run for certain models. We report these changes below, along with full descriptions of tuning procedures and selected hyperparameters for each model below.

*B1. Latent Dirichlet Allocation*

For the decomposition, we use the default hyperparameters for LDA in scikit-learn. In combination with the linear SVM, then, there are 2 hyperparameters for our combined LDA+SVM classifier: the number of topics *n_topics_* for the LDA; and the C parameter for the SVM. We consider *n_topics_* in {5, 10, 15, 20, 30} and discrete values for C in {0.001, 0.01, 0.1, 1, 2, 8, 16}.. After grid search to minimize classification error on the validation set, the best value of *n_topics_* was 30, and the best value for C was 8.

*B2. Latent Semantic Analysis*

For the decomposition, we use the TruncatedSVD class in scikit-learn. In combination with the linear SVM, then, there are 2 hyperparameters for our combined LSA+SVM classifier: the dimensionality *d* of the singular value decomposition for the document-term matrix; and the C parameter for the SVM. We consider *d* in {10, 25, 50, 100, 200} and discrete values of C in {0.001, 0.01, 0.1, 1, 2, 8, 16}. After grid search to minimize classification error on the validation set, the best value for *d* was 100, and the best value for C was 0.001.

*B3. Multinomial Naïve Bayes*

For our multinomial naïve Bayes classifier, we use the MultinomialNB class from scikit-learn, and we searched for continuous values of the smoothing parameter alpha in [0.0001, 1.0]. After GPO (*n_iter_*=50) to minimize classification error on the validation set, the best value of alpha was 0.032683.

*B4. Linear SVM*

For our SVM, with use the LinearSVC class from scikit-learn, and we searched for discrete values of C in {0.0001, 0.001, 0.01, 0.1, 1, 2, 5}. Minimizing classification error on the validation set, both grid search and GPO (*n_iter_*=50) settled on 0.0001 as the best value. We also note here that applying TF-IDF weights to the bigram feature vectors did not improve classification accuracy.

*B5. Random forest*

For our random forest, we use the RandomForestClassifier class from scikit-learn with n_estimators set to 1,000 and the rest of the hyperparameters to their defaults. As in Maenner et al. 2016, we chose a different threshold for classification than 0.50. To select our threshold, we examined classification accuracy for cutoffs in (0.01, 0.99) in steps of 0.01 on the initial validation set, and chose 0.47, which produced the highest accuracy. We used this threshold for the rest of our experiments.

As in Maenner et al. 2016, we found feature selection to improve model performance, and we explored 2 procedures: recursive feature elimination (RFE); and non-recursive feature elimination (nRFE). For both procedures, we begin by fitting the model to the training data, and we create a new document-term matrix containing only the top 250 most important features from this initial fit (this is mainly for the sake of convenience; stepping the model down from the full 860,493 bigrams would take an enormous amount of time). Then, we use the elimination procedures to find the number of remaining features *n_top_* that produces the highest accuracy on the validation set. For RFE, this means stepping down from the trimmed set of 250 features in increments of 10, stopping once the specified number of features has been reached; and for nRFE, this means simply specifying the number of features to keep and discarding all of the others. We allowed both methods to search for *n_top_* between 10 and 200 in steps of 10 (so possible values were 10, 20, 30, etc.). After this process, RFE chose a value for *n_top_* of 120, and nRFE a value of 130. To evaluate these two procedures, we tested each across the 10 train-test splits in the main experiment. The performance was similar, but nRFE posted the higher mean accuracy (87.10 vs. 86.83), and so its results are what we report.

For all the random forest models, we used TF-IDF-weighted count-valued bigram feature vectors as inputs.

*B6. NB-SVM*

We formulate our NB-SVM in the same way as Wang and Manning (2012), i.e. by converting binarized (where any count > 1 is converted to 1) bigram features to NB features and then allowing for interpolation between these and the SVM. For the SVM, we use the LinearSVC class from scikit-learn, and we modify it with custom code in NumPy. Like Wang and Manning, we keep squared hinge loss and the L2 penalty for the SVM, and we use a value of 1.0 for the NB smoothing parameter α. Thus, there are only two hyperparameters to tune here: the C parameter for the SVM, and the interpolation parameter β. We search for continuous values of β in (0.0, 1.0) and discrete values of C in {0.001, 0.01, 1.0, 2, 4} (we note that Wang and Manning use β=0.25 and C=1.0). After GPO (*n_iter_*=30) to minimize classification error on the validation set, we settled on β of 1.0 and C of 0.001, which is equivalent to an SVM with NB features and no interpolation

*B7. Neural networks*

We formulate our neural networks as deep averaging classifiers where the first layer is a lookup matrix for the feature embeddings, and the second is a fully-connected layer with a sigmoid activation. In our first model NN_sum_, the word embeddings are added together before being passed to the second layer, and in our second model NN_avg_, they are averaged (the latter approach is used by the fastText classifier). In both models, we also apply dropout to the output of the embedding layer to prevent overfitting. For training, the networks were optimized using Adam (Kingma 2014) to minimize binary cross-entropy loss on the training data until loss on the test data had not increased for some a fixed number of epochs. This number, which we call the patience parameter, was treated as a hyperparameter and tuned along with several other hyperparameters using the procedures described below.

As our hyperparameters, we considered dropout probability *p*, patience, embedding size *e*, minibatch size, and learning rate. For NN_avg­_, we searched for continuous *p* in (0.0, 0.9), discrete patience in {5, 10, 15, 20, 25}, discrete embedding size in {64, 128, 256, 512}, discrete minibatch size in {32, 64, 128, 256}, and discrete learning rate in (0.0001, 0.001, 0.01, 0.1); for NN_sum_ the ranges for the same parameters were (0.0, 0.9), {2, 5, 10}, {64, 128, 256, 512}, {32, 64, 128, 256}, and {0.00001, 0.0001, 0.001}, respectively (this model was more likely to overfit, so we lowered the learning rate and decreased patience accordingly). After GPO(*n_iter_*=20), the optimal hyperparameters for NN_avg_ were {0.75, 10, 64, 32, 0.001}, and the optimal hyperparameters for NN_sum­_ were {0.86, 5, 64, 256, 0.00001}.

Both models were trained on unigrams only and not bigrams; including the latter did not improve performance.

**REFERENCES**

Kingma DP, Ba J. Adam: A method for stochastic optimization. arXiv preprint arXiv:1412.6980. 2014 Dec 22.
